# Incidence of Acanthamoeba Keratitis in Switzerland

**DOI:** 10.3390/microorganisms13092032

**Published:** 2025-08-30

**Authors:** Frank Blaser, Felix Grimm, Philipp B. Baenninger, Zisis Gatzioufas, Michael A. Thiel, Moreno Menghini, Beatrice E. Frueh, Konrad Muehlethaler, Marco Alder, Kattayoon Hashemi, René Brouillet, Horace Massa, Manolito L. Finger, Christoph Tappeiner, Anthia Papazoglou, Florentina Joyce Freiberg, Gilbert Greub, Daniel Barthelmes, Sandrine A. Zweifel, Sadiq Said

**Affiliations:** 1 Department of Ophthalmology, University Hospital Zurich, University of Zurich, 8091 Zurich, Switzerlandsaidsadiq@gmx.ch (S.S.); 2 Institute of Parasitology, University of Zurich, 8006 Zurich, Switzerland; 3 Department of Ophthalmology, Cantonal Hospital Winterthur, 8400 Winterthur, Switzerland; 4 Department of Ophthalmology, University Hospital Basel, 4056 Basel, Switzerland; 5 Department of Ophthalmology, Cantonal Hospital Lucerne, 6000 Lucerne, Switzerland; 6 Department of Ophthalmology, Institute of Clinical Neurosciences of Southern Switzerland (INSI), Ente Ospedaliero Cantonale (EOC), 6900 Lugano, Switzerland; 7 Department of Ophthalmology, Bern University Hospital, Inselspital, University of Bern, 3010 Bern, Switzerland; 8 Institute for Infectious Diseases, University of Bern, 3012 Bern, Switzerland; 9 Department of Ophthalmology, Cantonal Hospital St. Gallen, 9007 St. Gallen, Switzerland; 10 Department of Ophthalmology, University of Lausanne, Jules-Gonin Eye Hospital, Fondation Asile des Aveugles, 1004 Lausanne, Switzerland; 11 Institute of Microbiology, University of Lausanne, 1015 Lausanne, Switzerland; 12 Department of Ophthalmology, University Hospital Geneva, University of Geneva, 1205 Geneva, Switzerland; 13 Department of Ophthalmology, Cantonal Hospital Fribourg, 1752 Fribourg, Switzerland; 14 Department of Ophthalmology, Pallas Klinik, 4600 Olten, Switzerland; 15 Department of Ophthalmology, University Hospital Essen, University Duisburg-Essen, 45147 Essen, Germany; 16 Medical Faculty, University of Bern, 3012 Bern, Switzerland; 17 Department of Ophthalmology, Cantonal Hospital Aarau, 5000 Aarau, Switzerland; 18 Department of Ophthalmology, City Hospital Triemli, 8055 Zurich, Switzerland; 19 Service of Microbiology, University Hospital of Lausanne, 1005 Lausanne, Switzerland; 20 Augenklinik Wettingen, 5430 Wettingen, Switzerland

**Keywords:** acanthamoeba keratitis, infectious keratitis, incidence, epidemiology, Switzerland

## Abstract

Despite rising global reports of Acanthamoeba keratitis (AK), the incidence of AK in Switzerland remains unknown. This investigator-initiated, retrospective, multicenter study assessed the nationwide incidence of PCR- and/or culture-positive Acanthamoeba results from January 2010 to December 2023. Data were collected from all tertiary care and large ophthalmological facilities in Switzerland, fully anonymized, and aggregated by month and year. We considered all corneal scraping results, whereby the detection method was specific to local standards. We identified 271 PCR- or culture-positive Acanthamoeba cases over 14 years. Applying the population data from the Federal Statistical Office in Switzerland, this corresponds to a mean incidence of 2.29 cases per million people annually. Infections were most common in summer (87 cases, 32.1%), followed by autumn (74 cases, 27.3%), spring (60 cases, 22.1%), and winter (50 cases, 18.5%). We found no significant change in incidence across the investigated period, *p* = 0.47. This nationwide study reveals a low but stable incidence of AK in Switzerland, in line with other industrialized countries but well below levels reported in tropical or densely populated regions such as India or Egypt. Seasonal variation supports the influence of environmental exposure and underscores the importance of preventive measures during warmer months.

## 1. Introduction

The genus Acanthamoeba comprises unicellular, opportunistic protists, which ophthalmologists know particularly as the cause of sight-threatening corneal infections [1,2]. As Acanthamoebae can tolerate a wide range of climatic circumstances, these free-living organisms are found worldwide but prefer soil and water niches [3,4]. Naginton et al. reported the first case of Acanthamoeba keratitis (AK) in 1974 [5], whereby the incidence increased with the higher use of contact lenses [6]. Since then, ocular trauma and contact lenses have been recognized as the leading risk factors for corneal infection [2]. Moist environments like cooling systems, humidifiers, showers, or sinks particularly enable the contamination of contact lenses [7]. Acanthamoebae exhibit a dual life cycle whereby they survive in unfavorable living conditions as dormant cysts [8]. They actively invade host tissues and exert pathogenic effects as trophozoites [8]. However, the versatile life cycle and the remarkable adaptability complicate the species classification based on immunological or physiological behavior [9]. Currently, DNA sequencing is used to differentiate Acanthamoeba species, identifying 23 distinct genotypes based on variations in their 18S rRNA sequences [10,11]. The clinical significance of Acanthamoeba keratitis (AK) lies in the severe infections and its capacity for endosymbiosis, harboring other pathogenic microorganisms and supporting their pathogenicity mechanisms [11,12].

Corneal opacities remain a major public ophthalmic health problem and are classified by the WHO as one of the leading causes of blindness worldwide [13]. Due to non-trachomatous corneal opacities, an estimated 5.5 million people aged 40 years or older globally suffer from bilateral moderate to severe vision impairment and blindness, and a further 6.2 million people have unilateral blindness [14]. However, the term “corneal blindness” conceals a potpourri of causes, including trauma, infection, immunological disorders, and congenital abnormalities. How many of these opacities are attributable to microbial infections is difficult to determine and has likely been conservatively estimated due to underreporting in financially disadvantaged countries. Although bacterial keratitis is significantly more common, AK cases are increasingly being reported, possibly due to the spread of contact lens use in industrialized countries [15]. A recent study by the University Hospital Zurich in Switzerland also mentioned the association of AK with contact lens use [16]. Nevertheless, the current understanding of its global epidemiology remains incomplete.

In Switzerland, the incidence of AK has not been investigated yet. Given the potentially sight-threatening corneal infection, knowledge of the incidence and possible increasing trends is essential. This retrospective study examines the nationwide incidence of AK confirmed by polymerase chain reaction (PCR) or culture. Understanding the incidence may help in prevention campaigns, early disease recognition, and resource allocation, which ultimately aim to improve patient outcomes.

## 2. Materials and Methods

This investigator-initiated, retrospective, multicenter study assessed the incidence of PCR or culture-positive Acanthamoeba results after corneal scrapings between January 2010 and December 2023. We included all tertiary care or large ophthalmological facilities in Switzerland, namely the University Hospital Zurich, the City Hospital Triemli, the Cantonal Hospital Winterthur, the Cantonal Hospital St. Gallen, the University Hospital Basel, the Bern University Hospital, the Cantonal Hospital Lucerne, the Cantonal Hospital Fribourg, the Pallas Kliniken, the Cantonal Hospital Aarau, the Ticino Cantonal Hospital, the Jules-Gonin Eye Hospital, and the University Hospital Geneva. The leading ethics committee in Zurich waived our study protocol, as it does not fall within the scope of the Human Research Act (BASEC numbers 2024-00847 and 2023-01146). The responsible committees in Basel (for the hospitals in the cantons of Aargau, Basel, and Lucerne), Bern, Vaud, Geneva, St. Gallen, and Ticino followed the ethical assessment of Zurich, hence ensuring that all centers included in this study are considered. We handled all data according to Good Clinical Practice guidelines.

### 2.1. Data Collection

We first identified the largest ophthalmology facilities in Switzerland and the associated laboratory establishments. After agreeing to participate in the nationwide study, we examined the electronic medical records for culture and PCR-positive Acanthamoeba results. We considered all corneal scrapings in patients with suspected infectious keratitis, without including the contact lens assessments. The corresponding author collected and compiled all data, whereby the retrieval was completely anonymized so that we only received the number of positive cases per month and corresponding year without any further patient information. In cases of several positive results from one patient, only one was counted to not falsify or overestimate the final incidence. We defined incidence as the number of new cases per year and relied on the yearly population values published by the Federal Statistical Office [17].

### 2.2. Acanthamoeba Detection

The same laboratory evaluates the corneal scrapings for possible Acanthamoeba infections in the cantons of Zurich, Aargau, Lucerne, and St. Gallen, whereby the used PCR methodology has been described in a previously published manuscript [16] and is briefly repeated here. Sample preparation and DNA extraction: liquid samples containing corneal scrapings were centrifuged (3 min, 14,000× *g*), and the ‘pellet’ (although invisible in most cases) together with 200 microliters (μL) of the supernatant was used for DNA isolation in 1.5 milliliter (mL) Eppendorf tubes. If samples contained a ‘corneal brush’, the brush was cut into two small pieces and added to the fluid. DNA was isolated from all sample types using the tissue protocol of the QIAamp DNA mini kit (Qiagen, Hilden, Germany) according to the manufacturer’s instructions. After the proteinase K digestion step, samples containing solid particles were centrifuged (1 min, 14,000× *g*), and the supernatant was used for the following steps. A sample of 5 µL of the final DNA eluate (DNA concentrations of 6.0–22.4 ng/µL, estimated using a NanoDrop One spectrophotometer, Thermo Fisher Scientific, Zurich, Switzerland) was used per reaction. DNA amplification: The primers and TaqMan probe used to amplify part (105 bp) of the 18S rRNA gene were Aca-F1 (5′-CCC AGA TCG TTT ACC GTG A-3′) [18], Aca-R1 (5′-GAG GAC AGG GTC CTA TTC CA-3′), and Aca-S1 (5′-FAM-TTC TGC CAC CGA ATA CAT TAG CAT GGG ATA-BHQ1-3′), all synthesized by Microsynth (Microsynth AG, Balgach, Switzerland). Primers were analyzed using the online OligoAnalyzer tool (Integrated DNA Technologies, Coralville, IA, USA). The risk of self- or hetero-dimer formation was not identified. Samples were tested in duplicate in a reaction volume of 25 μL. TaqMan Fast Advanced Master Mix (applied biosystems by Thermo Fisher Scientific, Zurich, Switzerland) was used with 900 nM of each primer and 200 nM of probe. In silico analysis showed that Acanthamoeba genotypes T1 to T15 were recognized. This included genotype T4, which is most frequently found in Acanthamoeba keratitis, as well as the rarer keratitis genotypes T2, T3, T5, T6, T8, T9, T11, T13, and T15 [19]. Serial dilution experiments using Acanthamoeba castellanii, strain 2HH (genotype T4), grown in vitro showed that positive reactions can be expected with a DNA amount equivalent to 1–5 cells per reaction. No reaction with genomic DNA of Balamuthia, Naegleria (both kindly provided by N. Müller, Bern, Switzerland), Hartmanella (kindly provided by J. Walochnik, Vienna, Austria), Enterocytozoon, Encephalitozoon, Entamoeba, Toxoplasma, Leishmania, Plasmodium, Trypanosoma, or Babesia was observed. Amplifications were run in a 7900HT Fast Real-Time PCR System (Applied Biosystems by Thermo Fisher Scientific, Waltham, MA, USA) under standard conditions (2 min at 50 °C, 10 min at 95 °C, and 45 cycles of 15 s at 95 °C and 1 min at 60 °C). All samples were tested in duplicate. If both reactions gave positive signals at fewer than 38 cycles, samples were judged as positive. Weak positivity means that the positive signals were detected between 38 and 42 cycles. Positive and negative (no template) controls were included in all runs. The absence of potential inhibitory effects and the amplification efficiency were monitored by adding 5 μL DNA eluate to an additional reaction amplifying a part of the 18S rRNA gene of Phytophthora citricola, a plant pathogen.

The University Hospital Geneva and the Jules-Gonin Eye Hospital share the same laboratory in Lausanne regarding the Acanthamoeba PCR assessments. Samples are generally received as smears in a liquid storage medium (1 mL) or liquid format. The sample is aliquoted into two 2 mL tubes: A first tube for DNA extraction, containing a volume of 0.25 mL, and the rest of the sample is kept in a second tube for archiving. Prior to extraction, the 250 µL sample undergoes lysis pretreatment by adding 180 µL ATL buffer and 20 µL proteinase K, followed by incubation at 56 °C for a minimum of 1 h and a maximum of 3 h. Next, 200 µL of PBS buffer is added to complete the pretreatment step. Samples are extracted with the MagNA Pure 96 extractor from Roche Diagnostics^®^ (Roche Diagnostics GmBH, Mannheim, Germany), using the MagNA Pure 96 DNA and Viral NA—Small Volume Kit (Ref. 06543588001: Roche Diagnostics^®^) and the Pathogen Universal 200 v4.0 protocol. During extraction, 20 µL of commercial internal control is added. This is the DNA Process Control Kit (Ref. 07339542001: Roche Diagnostics^®^). The extraction volume is 200 µL, with a final elution of 100 µL. DNA amplification is performed using TaqMan technology. Primers used are Primer F (5′-ATACCGTCGTAGTCTTAACCATAAACG-3′), Primer R1 (5′-GCCGATGGTGGTGTTTTGTATT-3′), and Primer R2 (5′-CAAAGACTTGATGATTTCTCACAAGCT-3′), amplifying a 76 to 188 bp fragment of the 18S rRNA gene. Detection is ensured by a FAM/BHQ-1-labeled 5′-TGCCGACCAGCGATTAGGAGACGTT-3′ probe. Primers and probes were synthesized by Eurogentec^®^. Eluates are tested in duplicate in a final reaction volume of 20 µL, made up of 10 µL TaqMan Fast Advanced Master Mix (Ref. 4444557: Applied Biosystems^®^), 5 µL primer and probe mix (with a final concentration of 200 nM for primers and 100 nM for probe), and 5 µL water (H_2_O). In parallel, each eluate is tested in simplicates with the Roche Diagnostics^®^ commercial internal control detection kit, according to a similar composition, where the primers/probe mixture is replaced by 5 µL of DNA Process Control Kit reaction mixture (Ref. 0733954200: Roche Diagnostics^®^). Amplification is carried out on a QuantStudio 7 Pro (Applied Biosystems, Massachusetts, USA) with a thermal program comprising an initial phase of 2 min at 50 °C, followed by activation for 10 min at 95 °C, then 45 cycles comprising denaturation for 1 s at 95 °C and hybridization/elongation for 20 s at 60 °C. Positive and negative controls accompany sample amplification to guarantee reliable results. Three dilutions of a plasmid specific to the PCR target, synthesized by RD-Biotech (Besancon, France), are used (1000, 100, and 10 copies per 5 µL). A negative control is also included, consisting of a 250 µL PBS sample, which has followed the same pre-analytical path as the tested samples. Results are read using Applied Biosystems Design and Analysis Software 2.6.0, enabling amplification curves to be assessed and visualized. Results are then interpreted using an in-house solution, incorporating decision-making algorithms considering Acanthamoeba PCR, Delta RN, cycle value, positivity observed in duplicates, and a PCR evaluation targeting the internal control validating the various processing stages. These different analysis points enable providing a result that meets quality criteria.

The Cantonal Hospital Fribourg and the University Hospital Basel send the corneal scrapings to the laboratory of the Bern University Hospital to analyze for possible Acanthamoeba infection. Cultural assessment was performed until the end of 2022, after which PCR testing was introduced as the standard. The PCR method was similar to the one described above. For cultural assessment, Page’s Saline Agar was inoculated with *E. coli* using a sterile cotton swab. *E. coli* serves as a food source for the Acanthamoeba. The Petri dish was sealed with Parafilm and incubated in a closed plastic dish (humid chamber) at +35 °C. The culture was read daily for 10 days under an inverted microscope at 100–200× magnification. Any Acanthamoeba present feed on *E. coli*, divide, and cover the agar plate within a few days. If the food is no longer sufficient, the Acanthamoeba trophozoites transform into cysts. When the amoebae grow on Page’s Saline Agar, typing is based on the morphology of the trophozoites and cysts. Trophozoites move extremely slowly over the agar surface. Contractile vacuoles can be observed. Acanthamoeba may be confused with bacteria or host cells. Hence, Wheatley’s trichrome stain for protozoa can be used for differentiation.

The Ticino Cantonal Hospital uses sample culture on a self-produced medium called “Page Agar” (salt solution and agar) for the detection of Acanthamoeba. An *E. coli* strain, ATCC 8739, was inoculated onto the medium from a very rich suspension by drawing a cross with a loop on the page agar medium. After three hours of incubation, the material (sediment after centrifugation of the phage solution with corneal scraping) was deposited on the phage agar medium at the intersection of the *E. coli* inoculum. The plate was subsequently incubated at room temperature for 14 days with readings every 24 h. Growth was detected by microscopy directly on the medium.

### 2.3. Statistical Analyses

Applying descriptive statistics, we present means with standard deviation, or minimum to maximum values for continuous data, and numbers and percentages for categorical data. We applied linear regression to explore trends in incidence over the years. We pre-defined the seasons as spring (March, April, and May), summer (June, July, and August), autumn (September, October, and November), and winter (December, January, and February). Using generalized mixed-effects Poisson model accounting for month-level count data, we investigated seasonal incidence rates using the season as a fixed factor and treating the center as a random factor. We adjusted the rate comparisons between the seasons for multiple comparisons using Tukey’s method in a simple random intercept model. All analyses were performed using R version 4.4.3 (R Foundation for Statistical Computing, Vienna, Austria).

## 3. Results

**Figure 1:**
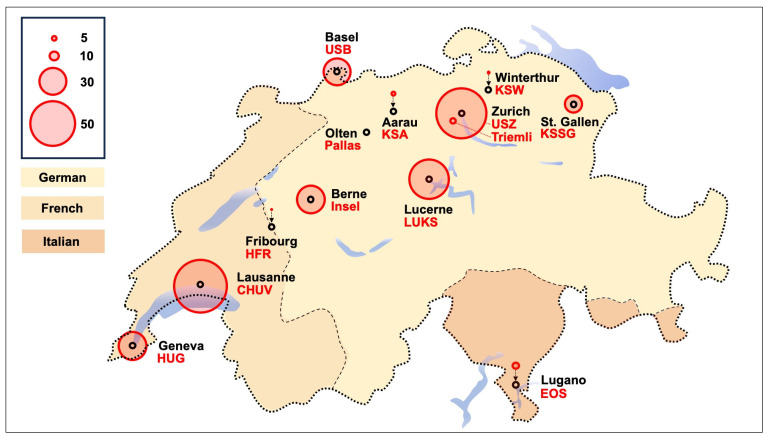
Illustration of the cumulative number of polymerase chain reaction or culture-positive Acanthamoeba cases reported per center on a map of Switzerland. The dashed line represents the linguistic relative separation between the country’s German, French, and Italian parts. The size of the red circle displays the number of positive Acanthamoeba cases in the final analyses across the studied time period from January 2010 to December 2023. EOS = Ticino Cantonal Hospital; HFR = Cantonal Hospital Fribourg; HUG = University Hospital Geneva; Insel = University Hospital Bern; CHUV = Lausanne University Hospital and Jules-Gonin Eye Hospital; KSA = Cantonal Hospital Aarau; KSSG = Cantonal Hospital St. Gallen; KSW = Cantonal Hospital Winterthur; LUKS = Cantonal Hospital Lucerne; Pallas = Pallas Kliniken; Triemli = City Hospital Triemli; USB = University Hospital Basel; USZ = University Hospital Zurich.

**Figure 2:**
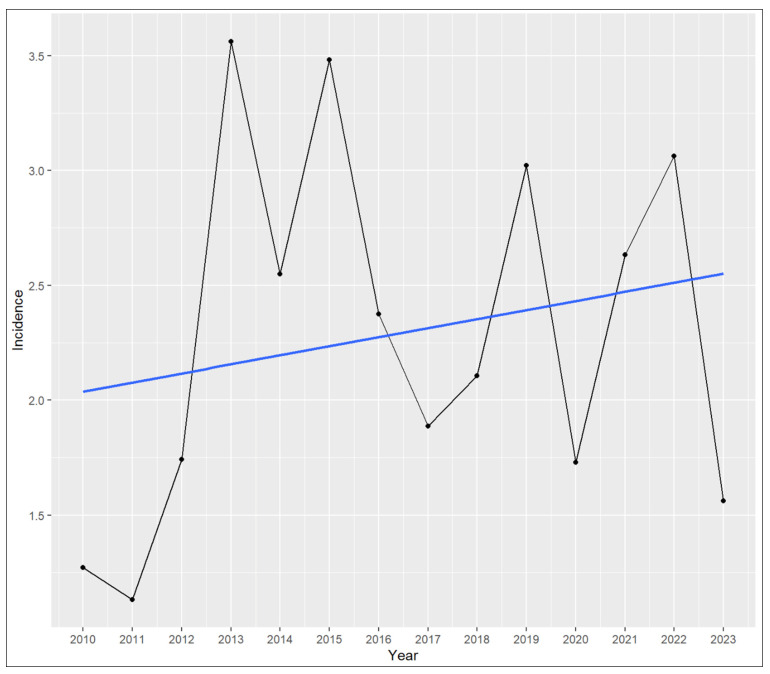
Annual incidence of positive Acanthamoeba results. The incidence rates are displayed as cases per million people. The blue line displays the regression trend line examined using a linear regression model. Although the data visually imply an increase over the years, linear regression analysis revealed no statistically significant temporal trend (estimate 0.039, standard error 0.053, t = 0.74, *p* = 0.47).

The analysis included 13 tertiary care centers and major eye clinics across Switzerland, which together provide treatment for AK. From January 2010 to December 2023, a total of 271 PCR- or culture-confirmed AK cases were reported. Based on annual Swiss population figures, this corresponds to a mean incidence of 2.29 cases per million people per year. The annual incidence varied over time, ranging from 1.13 cases per million in 2011 to a peak of 3.56 cases per million in 2013. Table 1 depicts the detailed incidence rates per year. Figure 1 illustrates the cumulative number of PCR- or culture-positive Acanthamoeba cases reported per center on a map of Switzerland. Linear regression analysis revealed no statistically significant temporal trend over the 14 years, *p* = 0.47. Figure 2 illustrates the annual incidence and regression trend line.

**Figure 3:**
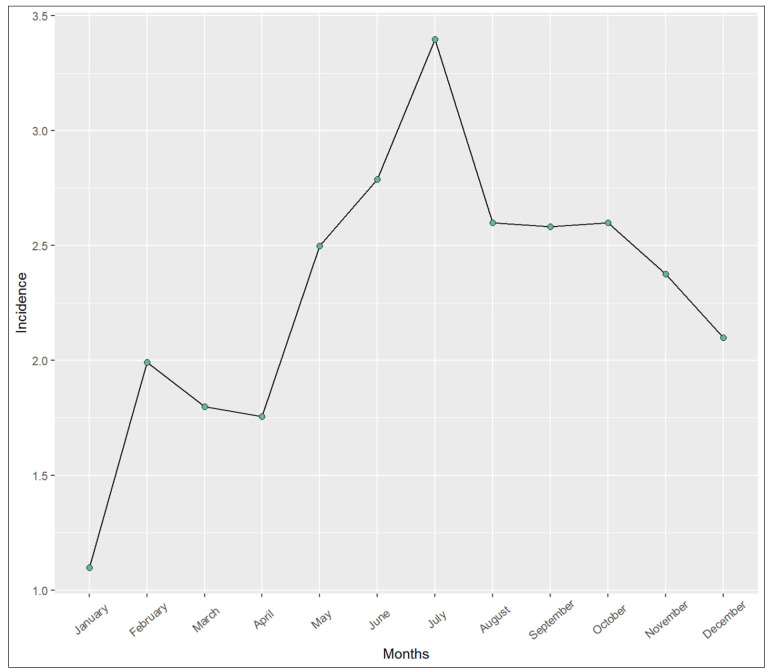
Seasonal case counts across the entire study period. The incidence rates are displayed as cases per million people. There was a significant difference in positive Acanthamoeba results between summer (June, July, August) and winter (December, January, February), *p* = 0.01. The other comparisons were not statistically significant.

Seasonal variation in case distribution was evident. The majority of cases occurred in summer (n = 87, 32.1%), followed by autumn (n = 74, 27.3%), spring (n = 60, 22.1%), and winter (n = 50, 18.5%). We applied a generalized mixed-effects Poisson model accounting for month-level count data to compare seasonal incidence rates. After adjusting for multiple comparisons using Tukey’s method, we found a statistically significant increase in summer case rates compared to winter, *p* = 0.01. No other seasonal comparisons reached statistical significance (autumn–spring *p* = 0.62, autumn–summer *p* = 0.74, autumn–winter *p* = 0.13, spring–summer *p* = 0.12, spring–winter *p* = 0.78). Figure 3 displays the seasonal case counts graphically.

Although we observed regional differences in the number of cases reported across participating centers, we did not conduct formal statistical analyses for inter-cantonal comparison. However, we calculated local incidences by respecting the linguistic relative separation of the referral patterns between the country’s German, French, and Italian parts. German-speaking Switzerland reported 189 cases of AK, French-speaking Switzerland 75 cases, and the Italian part 7 cases, corresponding to a local mean incidence of 2.25, 2.54, and 1.44 cases per million people per year, respectively. Table 2 presents the cumulative number of PCR- or culture-positive Acanthamoeba cases reported per center, and Table 3 provides an overview stratified per month.

## 4. Discussion

This nationwide, multicenter study reports the incidence of Acanthamoeba keratitis in Switzerland. Over a 14-year period, we identified 271 PCR- or culture-confirmed AK cases, corresponding to a mean annual incidence of 2.3 cases per million people. The incidence showed no statistically significant temporal trend throughout the study period. However, we found evident seasonal effects, with a significantly higher number of AK cases in summer than in winter. Our findings suggest a persistent disease burden, aligning with reports from other industrialized countries while remaining substantially lower than in tropical or densely populated regions [20].

With only a few national incidence studies regarding AK worldwide, our results complement the limited European literature. The first data from the UK in the 1990s reported an initial incidence of 1.4 cases per million, which increased in outbreak years associated with changes in the municipal water supply [21,22]. The latest data in the UK estimate an annual incidence of 2.35 cases per million [23]. However, other European countries like the Netherlands applied a different methodology to investigate the incidence, as they compared the number of AK cases to the total number of contact lens wearers [24]. In 2015, the reported incidence in the Netherlands was one AK case in 21,000 soft contact lens wearers, equivalent to a population-based incidence of 2.90 cases per million (considering a national population of 16,900,726 people) [24]. A systematic review and meta-analysis by Aiello et al. found an estimated international incidence of 2.34 AK cases per million people [25]. While some countries like Israel found a lower rate of 0.58 cases per million per year, others reported much higher numbers, like India with 15.2 cases, or New Zealand and Egypt with 5.2 and 5.0 annual cases per million, respectively [20,26,27]. These considerable differences in the incidence rates may be due to the different environmental and climatic conditions, water hardness and municipal water treatment systems, different contact lens use patterns and lens hygiene practices, clinician awareness and diagnostic capabilities (which may lead to mis- or underdiagnosis), and healthcare access or referral patterns.

Seasonal variation was a consistent and significant finding in our analysis, with the disease burden being higher in the summer and the number of cases significantly higher than in the winter months. Prior studies from England, Hong Kong, and Canada have corroborated this pattern [21,28,29]. Acanthamoeba thrives in warm conditions, with the optimal excystation temperature in vitro being 30 °C [30]. Together with the increased recreational activities like swimming and outdoor activities, this may explain the seasonal trend. Further, seasonality may partly reflect exposures occurring abroad. Swiss residents frequently travel to warmer regions during winter, where swimming in contaminated water sources and using substandard contact lens solutions abroad may occur. Hence, this travel activity could blur the true difference between the winter and warmer months in Switzerland, which may be more pronounced in reality. Although we lacked access to the patients’ travel histories, this hypothesis is consistent with prior observations linking travel with Acanthamoeba exposure [31].

Switzerland has a comparably high availability of ophthalmologists per capita [32], leading us to assume that the number of missed cases in this nationwide study is low. Nonetheless, our incidence estimate still likely underrepresents the true burden of AK in Switzerland. Firstly, we only included PCR- or culture-confirmed cases. False-negative results may have excluded clinically suspected cases, especially in culture, due to their lower sensitivity than PCR [33,34]. Secondly, smaller hospitals and private practices were not part of this multicenter cohort, whereby AK cases in Switzerland are almost exclusively treated in tertiary care centers. Lastly, clinical diagnoses and/or those by in vivo confocal microscopy are not represented in our study cohort. Consequently, our estimate of 2.29 cases per million people per year should be interpreted as a conservative or minimum incidence. Additionally, we did not perform a detailed regional analysis besides investigating the national linguistic separation. We hypothesize that the small cohort of the country’s Italian part, with an estimated population of 0.35 million people (around 4% of the country), and possible underreporting due to the geographically related (mountainous landscape) seeking of medical treatment in other Swiss cantons may explain the lower local incidence compared to the German or French parts. Moreover, although the case distribution varied across the different centers, overlapping referral regions and Switzerland’s nationwide health insurance system complicate geographic attribution. Patients are not restricted to cantonal healthcare providers and may receive care across administrative boundaries. Given the relatively small number of annual cases and the shared referral zones among large hospitals, any detailed regional breakdown would risk ecological misclassification and overinterpretation.

The strengths of this study include the complete inclusion of all tertiary ophthalmology institutions in Switzerland and the employed standardized, quality-controlled diagnostic methods. The 14-year study duration allows for robust estimation of temporal trends. Nevertheless, this study has some limitations. First, the retrospective design is inherently prone to selection bias and missing data. We analyzed monthly aggregated data rather than patient-level information, limiting our ability to assess patient demographics and individual risk factors such as contact lens type, hygiene practices, or exposure sources. Furthermore, due to the structure of the dataset, it was not possible to perform a month-to-month analysis over the study period without risking patient re-identification. Although monthly case numbers were available, they were not linked to the corresponding year. This prevented a more detailed temporal analysis that could have revealed short-term trends, year-to-year variability, or localized outbreaks. Further, we could not investigate co-infections, which are a considerable number in AK [16]. Future studies should incorporate more granular data to overcome the mentioned limitations. Lastly, diagnostic variability across centers, although minimized by adherence to validated protocols, may have influenced detection rates.

## 5. Conclusions

In summary, this nationwide multicenter study identified an annual incidence of 2.29 cases per million people. We found neither a significant increase nor a decrease in the incidence of AK over the study period from 2010 to 2023. Importantly, the reported incidence should be considered a minimum estimate, as false-negative microbiological results and purely clinical (non-microbiologically confirmed) cases were not represented in our dataset. The identified seasonal pattern emphasizes the need for heightened clinical vigilance during the summer when most cases occur. Understanding the epidemiology of AK is essential to guide prevention strategies, raise awareness among contact lens users, support early clinical recognition, and ensure adequate resource allocation. Ultimately, such measures aim to reduce the incidence and burden of this potentially blinding disease.

## Figures and Tables

**Table 1:** Detailed incidence rates per year. The population count is shown as count per thousand people. The incidence is depicted as cases per million people per year.

Year	Population Count (Per Thousand People)	Incidence (Cases Per Million People Per Year)
2010	7870	1.27
2011	7955	1.13
2012	8039	1.74
2013	8140	3.56
2014	8238	2.54
2015	8327	3.48
2016	8420	2.38
2017	8484	1.89
2018	8545	2.11
2019	8606	3.02
2020	8670	1.73
2021	8739	2.63
2022	8815	3.06
2023	8962	1.56
Overall mean	8415	2.29

**Table 2:** Cumulative number of polymerase chain reaction or culture-positive Acanthamoeba cases reported per center over the study period from January 2010 to December 2023.

Study Center	Swiss Canton	Total Number of Cases
Jules-Gonin Eye Hospital	Vaud	59
University Hospital Zurich	Zurich	53
Cantonal Hospital Lucerne	Lucerne	44
Bern University Hospital	Bern	31
University Hospital Basel	Basel	30
Cantonal Hospital St. Gallen	St. Gallen	19
University Hospital Geneva	Geneva	15
Ticino Cantonal Hospital	Ticino	7
City Hospital Triemli	Zurich	6
Cantonal Hospital Aarau	Aargau	4
Cantonal Hospital Winterthur	Zurich	2
Cantonal Hospital Fribourg	Fribourg	1
Pallas Kliniken	Solothurn	0

**Table 3:** Cumulative number of polymerase chain reaction or culture-positive Acanthamoeba cases stratified by month over the study period from January 2010 to December 2023.

Month	Number of Positive AK Cases
January	11
February	18
March	18
April	17
May	25
June	27
July	34
August	26
September	25
October	26
November	23
December	21
Total	271

## Data Availability

The original contributions presented in this study are included in the article. Further inquiries can be directed to the corresponding author.

## Abbreviations

The following abbreviations are used in this manuscript:

AK	Acanthamoeba keratitis
PCR	Polymerase chain reaction
